# The structural coloration of textile materials using self-assembled silica nanoparticles

**DOI:** 10.1007/s11051-017-3991-7

**Published:** 2017-08-31

**Authors:** Weihong Gao, Muriel Rigout, Huw Owens

**Affiliations:** 1 School of Fashion Technology, Shanghai University of Engineering Science, Shanghai, 201620 China; 2 School of Design, University of Leeds, Leeds, LS2 9JT UK; 3 School of Materials, The University of Manchester, Manchester, M13 9PL UK

**Keywords:** Structural coloration, Silica nanoparticles, Self-assembly, Photonic crystals, Artificial opal, Colloidal suspension

## Abstract

The work presented investigates how to produce structural colours on textile materials by applying a surface coating of silica nanoparticles (SNPs). Uniform SNPs with particle diameters in a controlled micron size range (207–350 nm) were synthesized using a Stöber-based solvent varying (SV) method which has been reported previously. Photonic crystals (PCs) were formed on the surface of a piece of textile fabric through a process of natural sedimentation self-assembly of the colloidal suspension containing uniform SNPs. Due to the uniformity and a particular diameter range of the prepared SNPs, structural colours were observed from the fabric surface due to the Bragg diffraction of white light with the ordered structure of the silica PCs. By varying the mean particle diameter, a wide range of spectral colours from red to blue were obtained. The comparison of structural colours on fabrics and on glasses suggests that a smooth substrate is critical when producing materials with high colour intensity and spatial uniformity. This work suggested a promising approach to colour textile materials without the need for traditional dyes and/or pigments.

**Figure:**
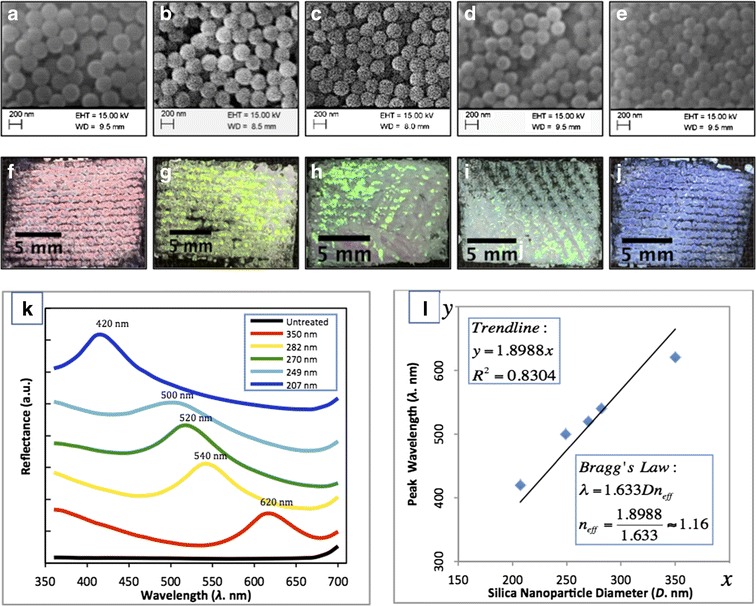
Graphical abstract

Graphical abstract

## Introduction

A photonic crystal (PC) is a periodic three-dimensional nanostructured system, which has the ability to control the propagation of light (Joannopoulos et al. 2011). Specifically, if the periodic nanomaterial is assembled from colloidal spheres, the PC is called a colloidal crystal (CC) (Meseguer 2005). Structural colour is one of the most important and interesting properties of PCs. The natural precious opal (Darragh et al. 1976), consists of a periodic nanostructure of highly ordered silica nanoparticles (SNPs), is probably the oldest and best-known example of PCs exhibiting structural colours. Inspired by natural opals, artificial opal PC materials have been fabricated using self-assembly (Marlow et al. 2009) methods: including sedimentation (Mayoral et al. 1997), vertical deposition (Jiang and Bertone 1999), physical confinement (Park et al. 1998), spin coating (Jiang and McFarland 2004) and dip-drawing (Liu et al. 2006). The optical properties of PCs have found use in several applications: optical switching (Kim et al. 2011), colour sensing and image display (Arsenault et al. 2007), security (Lee et al. 2013), novel pigments (Park et al. 2014), photonic papers and inks (Fudouzi and Xia 2003).

Compared to the surface colour from materials coloured with traditional dyes and pigments, structural colour has the advantages of high intensity, high resistance to fade, a play of colour effect, easy tunability of colours and less toxicity as the raw materials for producing structural colours are normally benign colloids such as silica or polymers (Zhao et al. 2012). Recently, there has been a growing interest (Finlayson et al. 2011; Diao et al. 2013; Liu et al. 2013; Zhou et al. 2013, 2014; Sun et al. 2015; Yuan et al. 2015) in producing textile materials exhibiting structural colours. Currently, structurally coloured fibres have been obtained by the coating of uniform colloids onto the surface of a prepared fibre (Liu et al. 2013; Zhou et al. 2013; Sun et al. 2015), the self-assembly of the colloids produced CCs on the fibre surface giving rise to vivid structural colours. Instead of using a single fibre as the substrate, the coating of colloids onto pieces of fabric produced structurally coloured fabrics (Diao et al. 2013; Zhou et al. 2014). Moreover, structurally coloured opal fibres have been produced directly from the self-assembly of uniform colloids into a cylindrical shape without a core fibre (Finlayson et al. 2011; Yuan et al. 2015), and structurally coloured fabrics have also been knitted using the prepared opal fibres (Finlayson et al. 2011).

The majority of research in this emerging area has focused on the self-assembly of polymer colloids, such as poly(methyl methacrylate) (PMMA) (Liu et al. 2013) and polystyrene (PS) spheres (Diao et al. 2013; Shao et al. 2014). In addition, core-shell colloids including poly(styrene-methacrylic acid) (P(St-MMA)) (Zhou et al. 2014), poly(styrene-methacrylic acid) (P(St-MMA-AA)) (Yuan et al. 2015) and polystyrene-allyl methacrylic-polyethylacrylate (PS-ALMA-PEA) (Finlayson et al. 2011) have been synthesized using PS as the core for the fabrication of structurally coloured textile materials. Due to the facile control of polymer size using soap-free emulsion polymerization, a full range of structural colours has been achieved on polyester fabric by applying a surface coating of different sized P(St-MMA) colloids through the self-assembly approach by using natural gravity sedimentation (Liu et al. 2015b), vertical deposition (Liu et al. 2015c) and interface–gravity joint self-assembly (Chai et al. 2017).

However, the application of silica colloids for the structural coloration of textile materials has rarely been reported. This is probably due to the difficulties in controlling the diameters of SNPs in a particular size range. It is known that a natural opal having the SNPs only in the diameter range of approximately 150 to 300 nm can give rise to structural colours (Darragh et al. 1976). Although the method of synthesizing uniform SNPs in the micron size range has been well studied (Stöber et al. 1968; Iler 1979) and large quantities of uniform SNPs samples can be bought from commercial sources (Xia et al. 2000), the SNPs in this particular size range for structural colours are less achievable. This is due to the difficulties in controlling the complex two-stage reaction during the Stöber process that consists of the hydrolysis of TEOS and the condensation of silicic acid. The difficulties in obtaining target SNPs result in limited colours being produced on the surface of textile materials such as glass fibres (Liu et al. 2011), polyester fabrics (Zhang et al. 2015) and silk fabrics (Li et al. 2017). In addition, little work has been reported that investigates structural colours on cotton fabrics using SNPs.

In this paper, structurally coloured textile cotton fabrics have been successfully fabricated by the surface coating of colloidal SNPs using a gravity sedimentation self-assembly method. By facile control of the particle diameter of the SNPs, a wide range of spectrum colours from red to blue has been obtained. The morphological and optical properties of structurally coloured fabric samples were characterised to explain the structural colour on the fabric. In addition, the structurally coloured samples were produced on glass substrates from the same SNPs batches for comparison purposes.

## Experimental

### Chemicals and materials

The chemical reagents used throughout the experiments are displayed as follows: the precursor alkoxide tetraethyl orthosilicate (TEOS) (99.0%) was purchased from Sigma-Aldrich Co., LLC; the catalyst ammonia (NH_3_, 25% in H_2_O) and the solvent ethanol (EtOH, 99.9%) were obtained from Fisher Scientific Co., Ltd., UK; the hydrolyzing agent distilled water (DW) (H_2_O, distilled by an USF-ELGA water purifier) was dispensed from the laboratory. All the chemicals were used as received without any further purification. Black woven cotton fabrics (0.034 g/cm^2^) were obtained directly from the Chemistry and Coloration Centre in the School of Materials at The University of Manchester, and they were used without any modification. Glassware such as specimen tubes and petri dishes were used for the colloidal SNPs to settle. Before use, the glassware was be cleaned in DW, acetone and EtOH, respectively, and then air-dried. A Gallenkamp brand hot-box laboratory oven was applied to give an elevated temperature in order to accelerate the colloidal self-assembly process.

### Synthesis of uniform SNPs

Colloidal suspensions containing uniform SNPs were prepared using a modified Stöber-based method as described in our previous work (Stöber et al. 1968; Gao et al. 2016a), where the hydrolysis and condensation of silicone alkoxide was catalysed by ammonia. In a typical procedure, a starting mixture solution containing 8 ml of ammonia, 47 ml of ethanol and 3 ml of DW was prepared in a 250-ml round-bottom flask under vigorous stirring. When the temperature of the mixture solution reached 60 °C, the TEOS (6 ml) was then added into the solution. The solution was stirred using a PTFE stirrer blade connected to an overhead motor for 2 h until the reaction completed. Fixing the volume amounts of TEOS/H_2_O/NH_3_ (25%) and varying the volume of solvent EtOH, i.e. the solvent varying (SV) method, uniform SNPs can be achieved in a controlled size range which are suitable for the creation of structural colour. Table 1 shows the specific recipes for preparing five colloidal suspension batches in order to achieve different sized SNPs.

**Table 1:** Recipes for the preparation of colloidal suspensions containing different sized SNPs

Recipe number	EtOH (ml)	TEOS (ml)	H_2_O (ml)	NH_3_, 25% in H_2_O (ml)	Temperature (°C)
a	47	6	3	8	60
b	53	6	3	8	60
c	60	6	3	8	60
d	67	6	3	8	60
e	73	6	3	8	60

### Self-assembly of SNPs into coloured opal PC films

Silica opal PC films were produced using the prepared uniform SNPs in suspensions through a process of sedimentation self-assembly (Mayoral et al. 1997). Specifically, 3 ml of samples from an unpurified silica suspension containing uniform SNPs was allowed to settle on a flat glass substrate (petri dish in this case) through sedimentation under the force of gravity. Once the sample solution was dried at an elevated temperature of 60 °C in an oven, a solid opal PC film of stacked SNPs was obtained. Due to the uniformity of the sphere shape and the diameter range of SNPs in the self-assembled structure, the resulting opal PC films diffract incident white light, giving rise to a range of spectral colours from violet to red.

### Fabrication of structurally coloured fabrics by coating of SNPs

Black woven cotton textile fabrics were successfully coloured by the gravity sedimentation self-assembly of uniform SNPs. A small piece of black woven cotton fabric was placed on the bottom of a specimen tube, and then 3 ml of silica suspension without any purification and/or modification was poured into the tube to cover the fabric. The specimen tube was carefully transferred into an oven with an isothermal temperature of 60 °C. The use of an elevated temperature accelerated the self-assembly and crystallization rate of the SNPs. As the solvent evaporated, SNPs were deposited onto the surface of the fabric with the aid of gravity force. Using this surface coating of uniform SNPs, structurally coloured cotton fabric was produced due to the ordered silica PC formed on the surface of the fabric. A series of structural colours over a wide spectrum was obtained by coating of uniform SNPs having different diameters.

### Characterisation of SNPs and coloured film/fabric samples

The morphological properties of the SNPs, opal films and coloured fabrics were examined using a Hitachi S-3000N scanning electron microscopy (SEM). It should be noted that as silica is a non-conductive material, coating the dried silica sample with a conductive layer (gold in this case) is an essential process for the SEM examination. The particle diameter and particle size distribution of the SNPs was determined using a Malvern Zetasizer Nano S dynamic light scattering (DLS) device. DLS is an effective way to measure the particle size directly from an original silica suspension without the need for additional sample preparation such as drying and coating. Individual particles were also examined using a Phillips CM20 transmission electron microscope (TEM) to clearly observe the shape and uniformity of the SNPs. The images of the coloured opal films and fabrics were captured using the digital camera of an iPhone 5s. The spectral reflectance of the fabric samples was measured using a Datacolor 650 spectrophotometer, and the chromaticity coordinates of both the SNP-coated fabric and SNP-coated glass samples were measured using a Konica-Minolta CS-200 Chromameter. The colourfastness of the samples to light was tested (ISO 105-B02: 2014) by exposing the samples to artificial daylight produced by a Xenon Light Fastness Tester (James Heal Co., Ltd.). Samples were graded from 0 (not resistant to light) to 8 (very light fast) using a standard blue wool scale, depending on the lightness difference between the samples and wool standards.

## Results and discussion

Structural colours were successfully produced on the fabric substrates through the self-assembly of SNPs. The coated fabrics were all graded 8 using the blue wool scale, indicating the excellent light fastness of the SNP-coated fabrics. The quality of the structural colours produced on the sample surface is mainly affected by the properties of silica particles and the self-assembly behaviour of SNPs on the fabric surface. Uniformity and size range of SNPs are the prerequisites to achieve a particular structural colour, while an ordered structure of self-assembled SNPs (PC) determines the appearance of the structural colour. The results of synthesized uniform SNPs are presented in the “Uniformity and size range of SNPs **”** section, and this is followed by an investigation of the surface morphology of silica PC on fabric substrate in the “Self-assembly behaviour of SNPs on glass and fabric substrates **”** section. The relationship between the physical properties of SNPs/PCs and optical properties of the structural colours on fabrics will be discussed in the “Optical properties of structurally coloured fabrics” section.

### Uniformity and size range of SNPs

**Fig. 1:**
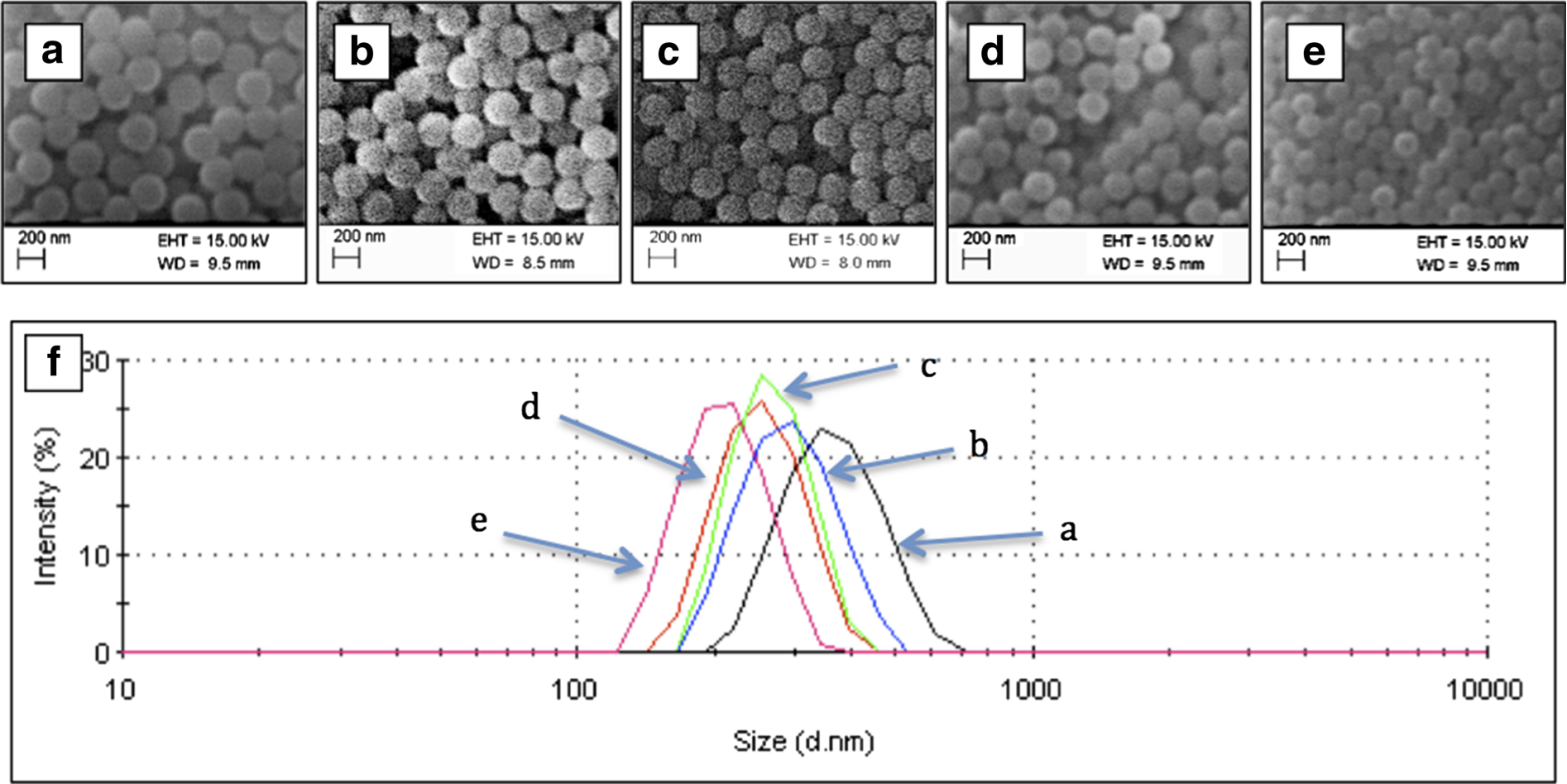
SEM images of SNPs prepared using EtOH volumes of 47 ml (**a**), 53 ml (**b**), 60 ml (**c**), 67 ml (**d**) and 73 ml (**e**), respectively; **f** shows the particle size distribution by intensity measured by DLS for samples (**a**)–(**e**)

**Fig. 2:**
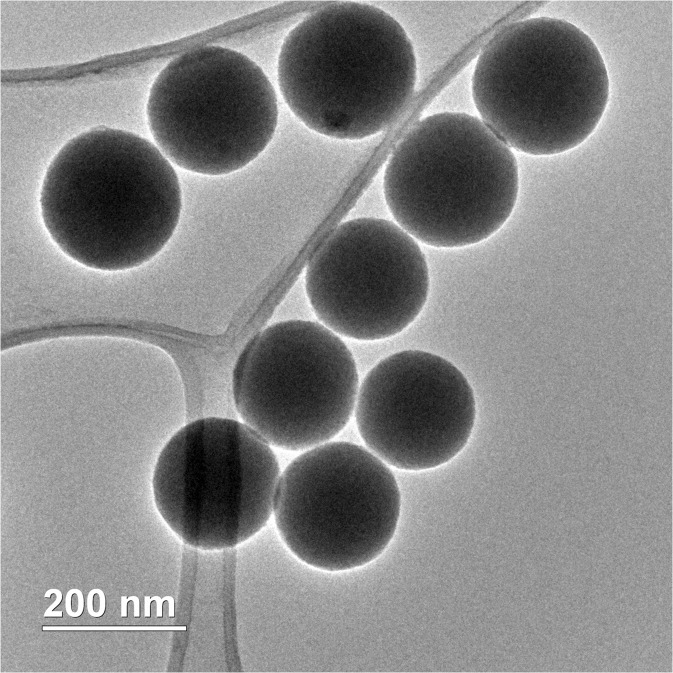
TEM image of SNPs prepared using EtOH volumes of 73 ml

Figure 1a–e shows the SEM images of SNPs prepared using the SV method with ethanol volumes of 47, 53, 60, 67 and 73 ml, respectively. These SEM images show that the SNPs are spherical in shape and uniform in size. Figure 1f describes the particle size distribution of SNPs measured using a DLS device. The peak of the distribution is narrow and sharp, which indicates that the prepared SNPs are uniform. The measured polydispersity index (PDI) for the five samples is 0.052, 0.016, 0.087, 0.025 and 0.020, respectively. The Malvern instrument Zetasizer manual (Malvern instruments 2004) states that a PDI smaller than 0.1 means that the samples are uniform and have a narrow size distribution. The PDI data confirms the uniformity of the prepared silica particles as their values are all below 0.1. One TEM image of SNPs prepared from the 73 ml EtOH batch is shown in Fig. 2, which also confirms the uniformity of the SNPs. The high-quality SNPs prepared in this work enabled the further fabrication of highly ordered PC structures on the fabric, although other researchers suggest that samples with PDIs smaller than 0.04 (Waterhouse and Waterland 2007) or 0.08 (Liu et al. 2015b) are considered to be uniform and suitable for the construction of PCs.

In our previous study (Gao et al. 2016a), a set of recipes for producing uniform populations of SNPs based on the Stöber method were outlined. This solvent varying (SV) method was used to prepare uniform SNPs in the diameter range of 70 to 400 nm. An exponential equation (Eq. 1) was proposed to predict the final particle diameter (*d*) from the initial solvent volume (*V*_EtOH_).

1 $$ d=885.45\exp \left(-0.02\left[{V}_{\mathrm{EtOH}}\right]\right) $$

**Fig. 3:**
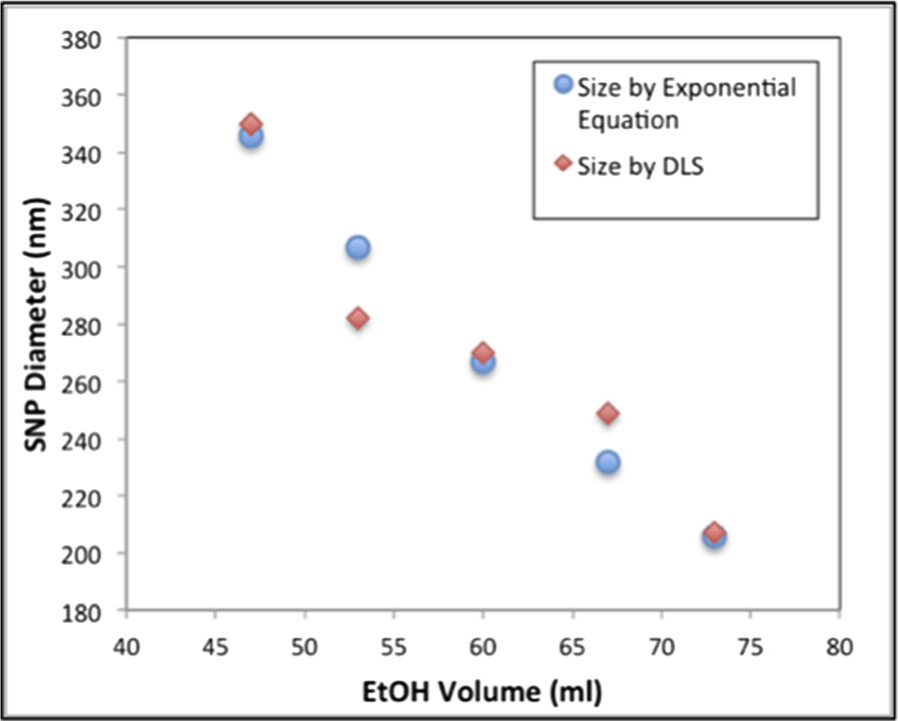
The average SNP diameter prediction from Eq. 1 (*circles*) and the DLS measured diameter in solution are plotted against the applied initial ethanol volume (*diamonds*)

Applying the SV method, five silica samples with different average diameters were produced by varying the initial solvent (EtOH) volume and fixing the other reaction conditions. The diameter of the SNPs in solution was measured using the DLS technique. Figure 1 shows the SEM micrographs of the five batches of SNPs of different target diameter and the size distribution by intensity from the DLS measurements. Figure 3 shows a plot of the initial EtOH volume against average measured SNP diameter and the predicted values from Eq. 1. The small deviation in the two sets of measured data confirmed the reproducibility and the reliability of the SV method for controlling the particle size.

### Self-assembly behaviour of SNPs on glass and fabric substrates

Through gravitational sedimentation, uniform SNPs were self-assembled on the surface of the glass and fabric substrates. The structure and self-assembly behaviour of the SNPs on the substrates were investigated by analysing SEM micrographs.

**Fig. 4:**
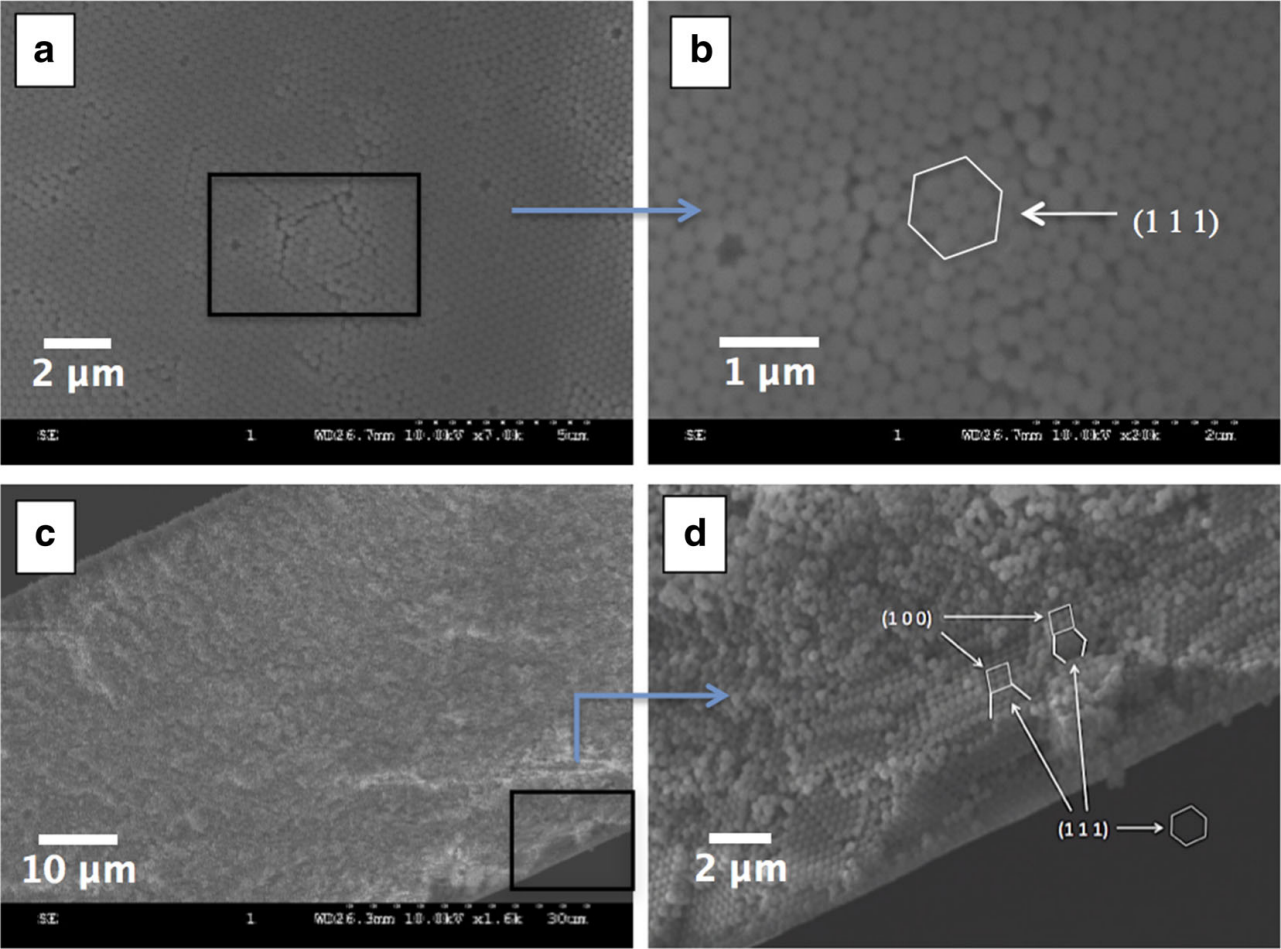
SEM images of an opal film prepared from 350 nm SNPs: the top view at a magnification of 7 K (**a**) and 20 K (**b**); a cross-sectional view is at a magnification of 1. 6 K (**c**) and an enlarged area of view from (**c**) that indicates the FCC structure (**d**). The scale bar is 2 μm, 1 μm, 10 μm and 2 μm for (**a**)–(**d**), respectively

Figure 4 shows the typical SEM micrographs of an opal film prepared on a glass petri dish, which self-assembled from a solution of uniform SNPs. Figure 4a is the top surface view of the opal film, and a magnified view of the surface in Fig. 4b shows the hexagonal close-packed arrangement of SNPs on the surface, which represents the (1 1 1) plane of the face-centered cubic (FCC) structure of the formed silica PC (Cheng et al. 1999). The cross-sectional view of the opal film is shown in Fig. 4c and a magnified area view is given in Fig. 4d. The square arrangement of SNPs in the cross-sectional plane can be observed in Fig. 4d, which represents the (1 0 0) plane of the FCC structure (Cheng et al. 1999). In addition, the hexagonal arrangement (parallel to the film surface) that is perpendicular to the (1 0 0) plane can be seen in Fig. 4d, this further proves the (1 1 1) plane of the FCC structure. Due to this ordered arrangement of uniform SNPs, a particular structural colour, or narrow bandwidth of reflected wavelengths, is produced by the Bragg diffraction of white light caused by the FCC structure of the opal film.

It should be noted that the opal film formed on the petri dish is uniform due to the smooth glass substrate, and this can be confirmed by the SEM micrographs of the film surface in Fig. 4a, b. However, textile substrates such as cotton fabrics have an uneven surface due to the complexity of the weave structure and roughness of the fibre material. Therefore, the SNPs cannot cover the entire surface of the fabric continuously resulting in non-uniform and discontinuous PC coatings. For example, some of the SNPs will fall into the voids between fibres and yarns, while other SNPs will locate on the prominent fibres and yarns forming thick silica blocks (PCs) with a FCC crystal structure. The different stacking behaviour of SNPs could give rise to different colour effects to be observed from the fabric.

**Fig. 5:**
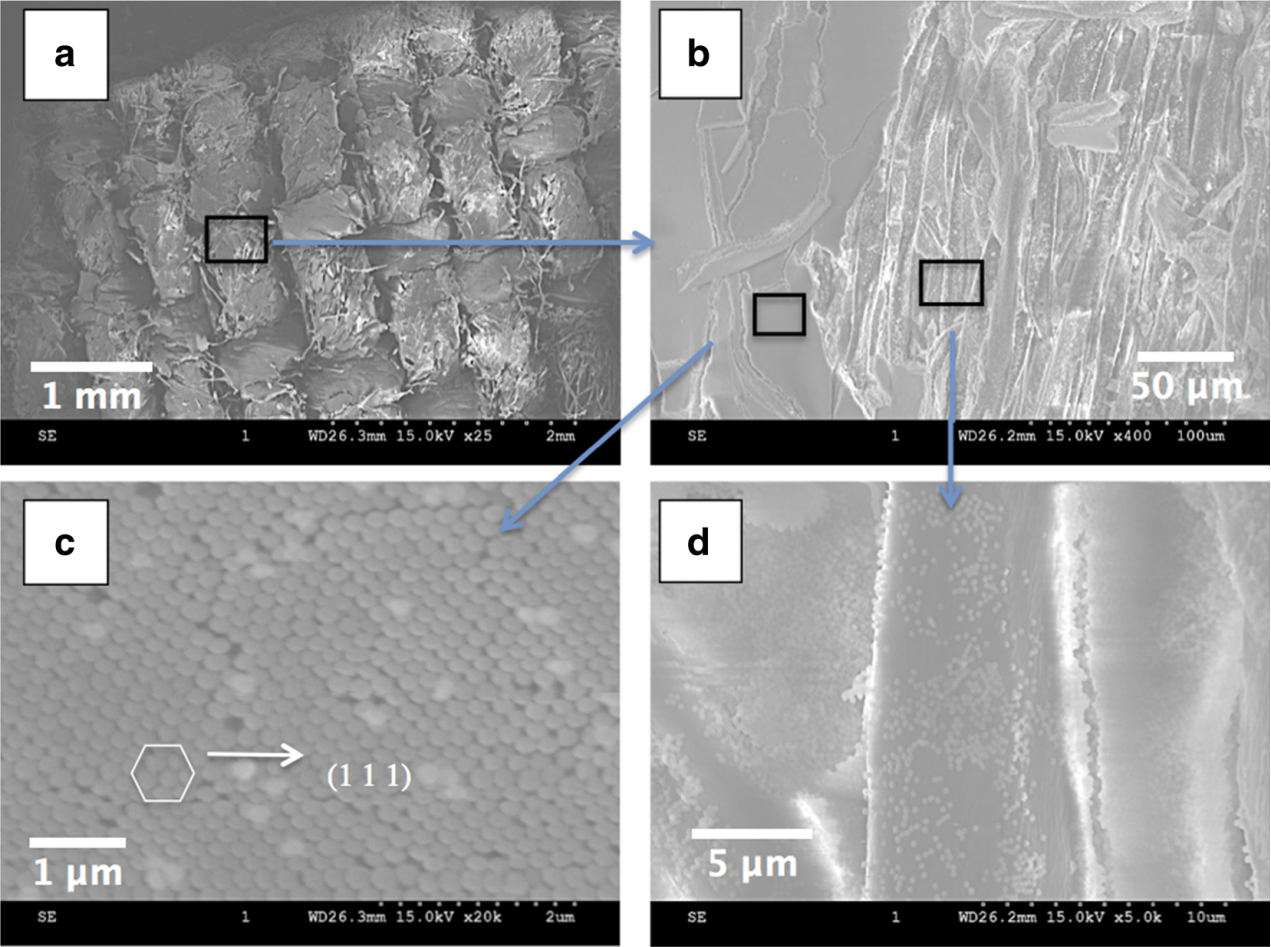
SEM images of a piece of woven cotton fabric coated with 282 nm SNPs. **a** Overview of SNPs-coated fabric. **b** Magnified area view of silica blocks and coated fibres. **c** Surface of silica thick block. **d** Fibre coated with SNPs. The magnification factors are 25, 400, 20 K and 5 K for (**a**)–(**d**), respectively. The scale bar is 1 mm, 50 μm, 1 μm and 5 μm for (**a**)–(**d**), respectively

Figure 5 shows SEM micrographs of a yellow coloured woven cotton fabric coated with 282 nm SNPs at different magnifications. At lower magnifications of 25 times, Fig. 5a, the woven structure of the fabric can be clearly viewed. It is noticeable that the PC did not cover the entire surface of the fabric as both silica blocks and individual floating yarns/fibres can been observed. Figure 4b shows a closer observation of the silica blocks and gaps at a magnification of 400, where it can be seen that there are large areas of smooth silica blocks on the left-hand side of the micrograph, and there are also some smaller isolated silica blocks on the surface of the yarns. The top view of the ordered structure of the silica block is shown in Fig. 5c, where the hexagonal arrangement of SNPs can be observed. Similar to the surface of opal films, this hexagonal arrangement corresponds to the (1 1 1) plane of the FCC structure. The top view of SEM micrographs provides some evidence for the FCC close-packed structure of PC that formed on the fabric (Liu et al. 2015b). In addition, Fig. 5d shows a magnified micrograph of SNPs-coated fibres in Fig. 5b, SNPs are attached to the surface of a fibre, but the particles are not close-packed and there is only one layer of particles. There will be no Bragg diffraction from such an area; thus, structural colours will not be generated. A white colour of silica particles or black background colour of the fibre can be possibly observed instead.

The self-assembly behaviour of SNPs on textiles substrates in this study is not in agreement with the work of Liu et al. (2015a), because they stated that the colloidal spheres will occupy the gaps of the fibres first and then start to stack on the actual surface of the fabrics, eventually forming a uniform PC covering the entire surface of the fabric. In this work, the SNPs were more likely to stack on the whole fabric surface including the yarn gaps when a colloidal suspension was subjected to gravity sedimentation so that an uneven coating of SNPs was obtained instead of a uniform PC coating. A reason for the different results could be the polyester fabric that they have used is more regular than the cotton fabric used in this case. Another reason could be the amount (5 ml) of colloidal suspension for sedimentation in their work is more than in this case (3 ml), so there were enough colloids to cover the entire surface of the polyester regardless of the roughness of the fabric.

### Optical properties of structurally coloured fabrics

Unsurprising, the opal film self-assembled on glass substrates from populations of uniform SNPs in a particular size range shows vivid structural colours due to the Bragg diffraction of white light caused by the ordered FCC structure of the PC. In our previous work (Gao et al. 2016b), structurally coloured silica opal colloidal crystal films have been fabricated, and it was found that the structural colour effect was tuned by the average diameter of SNPs and viewing angle. However, little has been reported about the structural colour properties of the silica PC on textile fabrics. In this work, a variety of structural colours were obtained by the self-assembly of SNPs on textiles fabrics and their optical properties determined. The relevant coloured SNPs opal films on glass will be provided for comparison purposes.

Although the self-assembly behaviour of SNPs on textiles substrates is more complex than one on a glass substrate, the ordered FCC structure of the PC can also be self-assembled on the surface of the fabric in terms of discontinuous thick silica blocks. Therefore, a non-uniform structural colour should also be observed from those individual small pieces of silica blocks in the same way that a continuous opal film diffracts white light to reflect a uniform structural colour. Moreover, a white colour could be seen from the coated areas with disordered SNPs or a thin layer of SNPs, and a black background colour will appear if the fibres/yarns were not coated with any SNPs at all. In addition, similar to the opal films on glass, the structural colour of the fabric could also be tuned by varying the particle size of the SNPs so that a wide range of visible spectral colours from red to blue could be achieved.

**Fig. 6:**
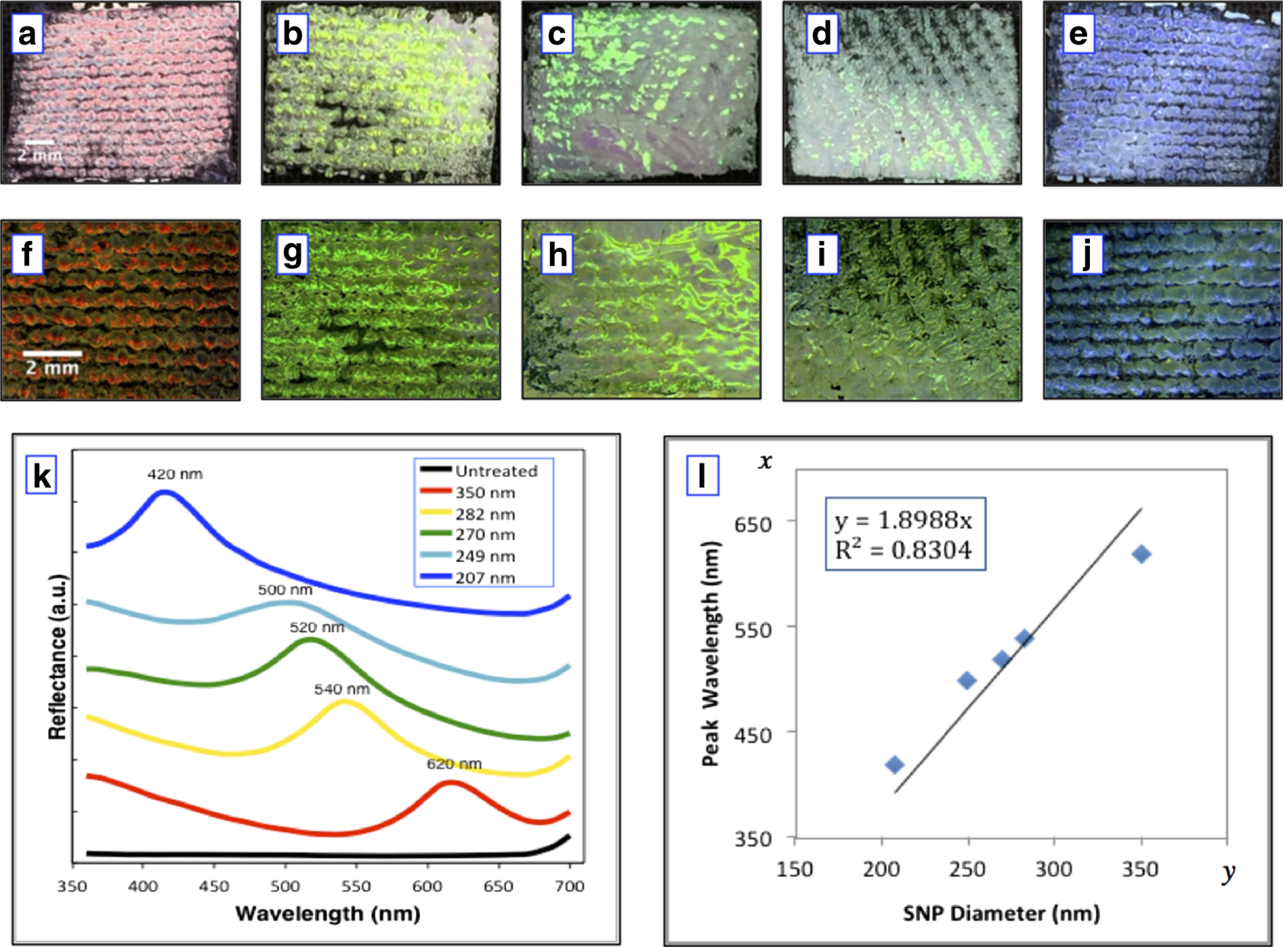
The images (**a**)–(**e**), microscopic images (**f**)–(**j**) and reflectance spectra (**k**) of structurally coloured woven cotton fabrics coated with SNPs having average diameters of 350 nm (**a** and **f**), 282 nm (**b** and **g**), 270 nm (**c** and **h**), 249 nm (**d** and **i**) and 207 nm (**e** and **j**), respectively; (**l**) shows a scatterplot where the particle diameter against peak wavelength of the structural colour is fitted with a linear function. The scale bars of (**a**)–(**e**) are the same and displayed in (**a**), the scale bars of (**f**)–(**j**) are the same and displayed in (**f**), both scale bars are 2 mm

Figure 6a–e shows the images of the structural colours observed on the woven cotton fabrics. The surface of the fabric is almost completely covered by the SNPs using the gravity sedimentation method. Figure 6f–j shows the magnified micrographs of those five coloured samples taken from a reflection microscope at a magnification factor of 7. The morphology and structure of the cotton fabric can be clearly observed from the images from the microscope. There are three different coloured regions on the fabric surface that can be distinguished: structurally coloured regions produced by the Bragg diffraction of white light produced by ordered thick silica blocks (PC), white coloured regions due to the scattering of white light due to a disordered or thin layer of SNPs and black coloured regions due to the absorption of white light by the black dyes of the background fabrics. Although the intense structural colour produced on the fabrics is not uniform, there exists a clear colour difference in terms of the hue property so that they all look different with the structural colour tuned by the change of the average diameter of SNPs. Visual inspection indicates that the structural colours for the five fabric samples in Fig. 6a–e are red, yellow, green, cyan and blue. These coated fabrics were produced by the gravity sedimentation and self-assembly of SNPs with average population diameters of 350, 282, 270, 249 and 207 nm, respectively. Figure 6k gives the spectral reflectance of the coloured fabrics obtained from a Datacolor 650 spectrophotometer. The position of the peak wavelength is the position of the photonic band gap (Liu et al. 2015b); the light is prohibited in this region and therefore to be reflected and seen by the observer. The colour of each curve is related to the fabric colour, and it can be seen that the peak reflectance values shift systematically in relation to particle diameter. The peak wavelengths of the structural colours have a red shift from 420 to 500, 520, 540 and 620 nm, when the particle size was increased from 207, 249, 270, 282 and 350 nm, respectively.

When viewing an opal material with a viewing angle of 0°, Bragg’s equation can be written as *λ* = 2*dn*_eff_, where *λ* is the peak wavelength of the reflectance of the opal material, *d* is the lattice spacing of (1 1 1) plane, *n*_eff_ is the effective refractive index of the opal material. Since *d* is equal to 1.633 times the sphere radius or 0.8165 times the sphere diameter *D*, Bragg’s equation becomes *λ* = 1.633*Dn*_eff_ (Tilley 2010). As for a particular material, *n*_eff_ is a positive constant number, so the peak wavelength *λ* will have a positive linear correlation with the particle diameter *D*. If the values of *λ* and *D* are known, the effective refractive index *n*_eff_ can be calculated. In Fig. 6l, the measured values of *λ* and *D* are plotted as *y* and *x* values, respectively. If a linear forecasting trend line is added to fit the scatterplot, the value of the slope of the linear function is equal to 1.633*n*_eff_ *.* It should be noted that if the sphere size was zero, there is no wavelength refection due to Bragg diffraction, so the trend line should cross the origin of coordinates. Thus, the trend line should have a zero intercept. The intercept was set to zero and a best-fit trend line was plotted producing a linear function of *y* = 1.8988*x* (slope is 1.8988) as displayed in the caption of Fig. 6l. From the slope (1.8988) of the trend line, the effective refractive index *n*_eff_ of the structurally coloured fabric is calculated as 1.16 (1.633*n*_eff_ = 1.8988).

**Fig. 7:**
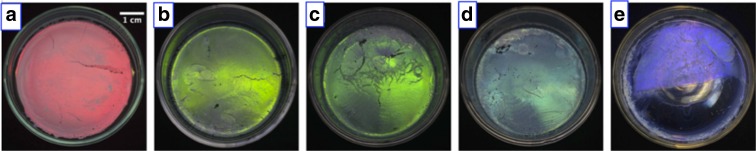
Structurally coloured silica opal films on glass prepared using SNPs with diameters of 350 nm (**a**), 282 nm (**b**), 270 nm (**c**), 249 nm (**d**) and 207 nm (**e**), respectively; the scale bar is 1 cm (Gao et al. 2016b)

**Fig. 8:**
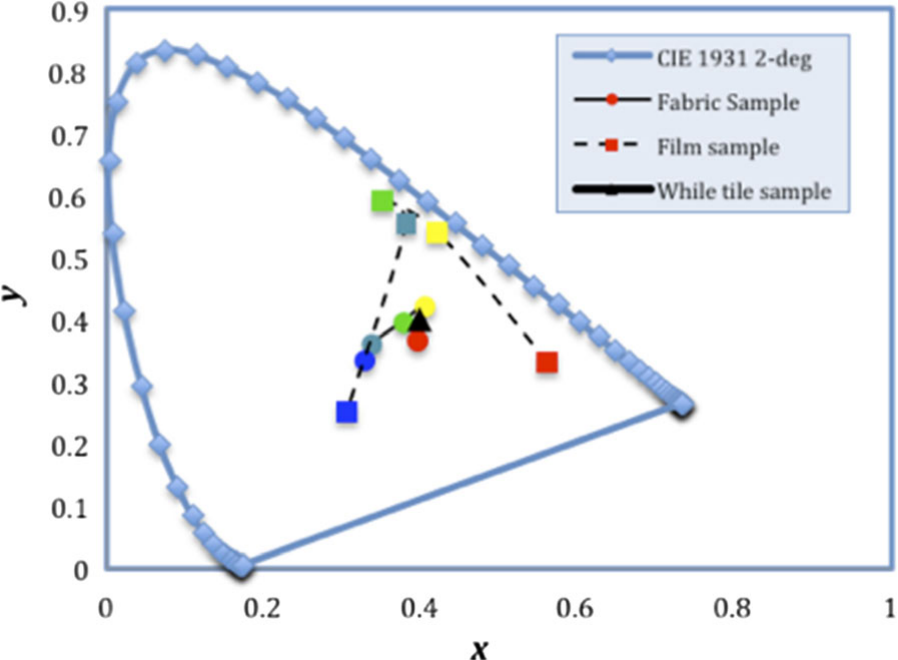
Chromaticity coordinates of five structurally coloured fabrics in Fig. 6a–e and five opal films in Fig. 7a–e; a white tile standard sample was also measured for comparison

It is necessary to clarify that the suspensions adopted for colouring these samples were prepared using the SV method; the volumes of the solvent ethanol were 47, 53, 60, 67 and 73 ml. Based on our previous work (Gao et al. 2016b), structurally coloured silica opal films, Fig. 7a–e, were successfully fabricated in petri dishes using the same silica suspensions prepared from the same recipes for producing the coloured fabrics in Fig. 6a–e. From these images of coloured samples, it can be seen that the coloured opal films on glass and the coated fabrics that were treated from the same batch of suspension present a similar hue property of red, yellow, green, cyan and blue, for recipes with ethanol volumes of 47, 53, 60, 63 and 67 ml, respectively. This can be explained by applying Bragg’s equation where the observed colour in terms of wavelength (*λ*) is only determined by the lattice spacing (*d*) or the sphere diameter (*D*) when the viewing angle and refractive index (*n*_eff_) are fixed. However, the opal film sample looks more vivid, bright and uniform, while the fabric samples are lighter, less saturated and less uniform. This difference can be observed more easily in Fig. 8, where the chromaticity coordinates of the film and coated fabric samples are plotted.

In Fig. 8, the *x* and *y* chromaticity coordinates of the film samples that have been connected with a dashed line are located closer to the horseshoe-shaped spectrum locus than those of the fabric samples within the highlighted oval area. This indicates that the film samples on glass have more saturated colours than the coated fabric samples, and this is in agreement with the visual observation. The reason for the difference of reflection between the films on glass and those on the fabrics probably arises from the surface morphology of the two different substrate materials. In general, textile fabrics are more complex in structure, with texture and uneven surfaces, and flexible when compared to the glass substrate. These factors make the self-assembly behaviour of SNPs more difficult to control. Therefore, the silica spheres can either self-assemble on top of the fabric’s surface to form thick silica blocks as shown earlier in Fig. 5 or pass through the gaps between the yarns/fibres contributing to defects of the as-formed PCs, resulting in a discontinuous silica coating and eventually leading to uneven structural colours on the fabric surface. It can be concluded that a flat surface such as a glass petri dish provides a homogeneous substrate for the SNPs to self-assemble. This allows the formation of high-quality PCs with fewer defects exhibiting intense and uniform structural colours.

## Conclusions

Structural colours have been successfully produced on textile substrates using the natural sedimentation and self-assembly of SNPs. Varying the particle diameter of the SNPs produces structural colours tuned over a wide visible spectrum from red, yellow, green and cyan to blue. Specifically, the structural colour produced will have a red shift with increasing particle diameter or a blue shift with decreasing particle diameter. The SNPs in the size range of 207 to 350 nm resulted in brilliant structural colours of blue to red with peak wavelengths of 420 to 620 nm. The comparison of structurally coloured opal films on glass and on fabrics suggests that a smooth substrate is critical when producing materials with high colour intensity and spatial uniformity. The fabric treated with SNPs was extremely light fast. This study highlights some of the issues associated with coating fabrics using a deposition method based on gravity sedimentation. Previous workers have suggested that the stacking of the SNPs in the voids of the fabric will produce uniform PCs. This work suggests that the uniform coating of fabric is more complicated. This strategy of colouring textile materials using environmental friendly silica is promising for textile coloration without the need for traditional dyes and/or pigments. The textile and fashion industries may benefit from the increased gamut and design possibilities afforded by this method.
